# Supplementary material for: Content-rich biological network constructed by mining PubMed abstracts
Source: BMC Bioinformatics. 2004 Oct 8;5:147. doi: 10.1186/1471-2105-5-147 (PMC528731; doi:10.1186/1471-2105-5-147)
Supplement: Additional File 2 — The original results of the above study (non-essential files are deleted to keep the file size under the limit set by BMC bioinformatics). [file 1471-2105-5-147-S2.bz2 › chilibotAdditionalFile2/dip05/330ID1328224E3926/html/TNF_TNFRSF1B.html]

 


 **TNF** and **TNFRSF1B** 
  
Found 687 abstracts in PubMed, retrieved 05.  
 

 What does Google say? 
 PDF only 
| .edu only 

---

**Interactive relationship** (e.g. stimulation, inhibition, etc)

**Neutral relationship**- LT consists of soluble LT alpha 3, which binds to  **TNF**  R1 and  **TNF**  R2  [ **TNFRSF1B** ] , and membrane LT alpha 1 beta 2, which binds to LT beta R.  Ref: 12882833 Int Immunol, 2003
- Inactivation of  **TNF**  alpha  [ **TNF** ]  and IL 1beta was achieved by repetitive intraperitoneal injection of etanercept  [ **TNFRSF1B** ]  and anakinra, respectively.  Ref: 12883478 HepatologyHepatology, 2003

**Non-interactive relationship** (e.g. studied together, co-existance, homology, etc.)

- This activity of LT depends on membrane LT alpha 1 beta 2 and not soluble LT alpha 3, since DA virus persisted and induced inflammatory demyelination in LT beta R, but not  **TNF**  R1 or  **TNF**  R2  [ **TNFRSF1B** ] , mice.  Ref: 12882833 Int Immunol, 2003
- In human hypertension HT plasma tumor necrosis factor  [ **TNF** ]   **TNF**  alpha  [ **TNF** ]  and soluble  **TNF**  receptor 2 fragment sTNF R2 are increased, and the  **TNF**  R2  [ **TNFRSF1B** ]  gene  **TNFRSF1B**  has been implicated.  Ref: 12878376 Am J Hypertens, 2003
- Etanercept  [ **TNFRSF1B** ] , a competitive inhibitor of  **TNF**  alpha  [ **TNF** ] , is currently FDA approved for psoriatic arthritis and rheumatoid arthritis.  Ref: 12894133 J Am Acad Dermatol, 2003
- Therefore, we measured Tnfr2  [ **TNFRSF1B** ]  mRNA in kidney, adrenal, heart, and aorta from rats with ACTH induced, corticosterone induced, and spontaneous HT SHR, and tested the effect of blockade of  **TNF**  alpha  [ **TNF** ]  by a recombinant  **TNF**  R2  [ **TNFRSF1B** ]  fragment huTNFR Fc on development of HT in the ACTH model.  Ref: 12878376 Am J Hypertens, 2003
